# Efficacy of systemic HS-198, an analogue of oxymorphone, on cancer pain-related behaviour in mice

**DOI:** 10.1186/1471-2210-11-S2-A4

**Published:** 2011-09-05

**Authors:** Muhammad F Asim, Catalina R Bohotin, Cristina E Constantin, Helmut Schmidhammer, Michaela Kress, Mariana Spetea

**Affiliations:** 1 Department of Pharmaceutical Chemistry, Institute of Pharmacy and Center for Molecular Biosciences, University of Innsbruck, 6020 Innsbruck, Austria; 2 Division of Physiology, Department of Physiology and Medical Physics, Innsbruck Medical University, 6020 Innsbruck, Austria

## Background

Cancer pain is a significant clinical problem being one of the first symptoms of disease with 75–90% of the patients experiencing chronic pain syndromes in advanced stages [1]. The management of cancer pain is mainly based on the use of opioid drugs; however their clinical use is limited by high incidence of adverse effects. There is a continued search for highly efficacious opioid analgesics with reduced complications and improved patient compliance. An analogue of the clinically used oxymorphone, 5-methyl-substituted 14-*O*-methyloxymorphone (HS-198), is a selective μ opioid agonist and a potent antinociceptive agent in animal models of nociceptive and inflammatory pain, while exhibiting a favourable dissociation between analgesia and the occurrence of side effects [2]. We report data on efficacy of this opioid agonist after subcutaneous administration (s.c.) in a murine model of cancer pain. The opioid receptor-mechanistic basis of the antinociceptive action was also investigated.

## Methods

Cancer pain was induced in C57BL/6J mice by s.c. implantation of lung carcinoma cells, in the plantar and dorsal side of the right hindpaw [3]. Mechanical sensitivity was determined using von Frey monofilaments. Heat sensitivity was assessed using the Hargreaves test. *In vitro* biological activities were evaluated using binding and functional assays.

## Results

On day 9 post-inoculation, s.c. HS-198 produced a dose-dependent inhibition with significant effects in attenuating cancer pain-related behaviour (thermal and mechanical hypersensitivity) on the tumour side. Pre-treatment with the opioid receptor antagonist naloxone reversed the antinociceptive effects induced by HS-198 in mice with cancer-induced pain. *In vitro*, HS-198 showed high affinity and selectivity for both mouse and rat μ opioid receptors, and it displayed potent μ-agonism through inhibition of G proteins.

## Conclusions

Systemic s.c. administration of the μ opioid receptor agonist HS-198 induces potent antinociceptive effects in mice with cancer pain via opioid receptor-specific mechanisms.
